# Parliament and the Profession

**Published:** 1917-05-26

**Authors:** 


### Parliament and the Profession.

### Supply of Nurses' Committee.

Copies of the Report of the Committee on Nurses for the War are now available for the use of members of the House of Commons.

### Proposed Military Venereal Hospital.

Mr. Macpherson stated that the question of making use of the military hospital huts at Cromarty as a hospital for the treatment of venereal diseases was under consideration.

### Venereal Diseases Bill.

A new clause penalising persons knowingly suffering from a venereal disease and failing to consult a medical practitioner was negatived. The Bill, as amended in Standing Committee, was considered, read for the third time, and passed.

### Medical Students.

Mr. Macpherson informed Mr. E. Harvey that fourth and fifth year medical students can be released, if on military service, to continue their studies; their employment with the Army is not considered desirable, and it is thought better to wait until they can be commissioned as medical officers.

### Combatant Medical Practitioners.

Mr. Macpherson announced that steps have been taken to withdraw medical practitioners who are officers of the fighting force. All medical practitioners who are known to be non-commissioned officers and men are invariably offered appointments as officers in the R.A.M.C. if fitted to hold His Majesty's Commission.

### Grants to Civilian Hospital Doctors.

Mr. Forster informed Sir William Collins that while the War Office gratefully receives the services of civilian doctors in voluntary aid hospitals, the General Officer Commanding has power under War Office instructions to allow suitable remuneration, in which case the grant to the hospital in question is increased by an equivalent amount.

### German Wounded on Hospital Ship.

Col. Lockwood, in a question, called attention to the fact that in a mixed convoy of English and German wounded twenty of the latter were given beds while some of the English had to lie upon the floor. Mr. Macpherson stated that the differentiation had nothing to do with nationality, but was in accordance with the condition of the patients—those in beds being cot cases and those on the floor slightly wounded.

### Medical Examination of Female Prisoners.

The Home Secretary, in answer to questions as to the circumstances under which two women were examined locally for venereal disease while under remand in Holloway Prison, stated that it appeared that the Brentwood magistrates had endorsed upon the committal order a statement that they would be obliged by the opinion of the medical officer as to whether the women were suffering from disease. Sir George Cave had informed the magistrates that, in his opinion, this request should not have been made, and the prison medical officers have definite instructions that no woman is to be examined without her consent, although in this case it was reported that the necessary consents were given.

